# The probability of chromatin to be at the nuclear lamina has no systematic effect on its transcription level in fruit flies

**DOI:** 10.1186/s13072-024-00528-8

**Published:** 2024-05-06

**Authors:** Alexander Y. Afanasyev, Yoonjin Kim, Igor S. Tolokh, Igor V. Sharakhov, Alexey V. Onufriev

**Affiliations:** 1 Department of Biomedical Engineering and Mechanics, Virginia Polytechnic Institute and State University, Blacksburg, VA 24061 USA; 2 Department of Computer Science, Virginia Polytechnic Institute and State University, Blacksburg, VA 24061 USA; 3 Department of Entomology, Virginia Polytechnic Institute and State University, Blacksburg, VA 24061 USA; 4 Department of Physics, Virginia Polytechnic Institute and State University, Blacksburg, VA 24061 USA; 5 Center for Soft Matter and Biological Physics, Virginia Polytechnic Institute and State University, Blacksburg, VA 24061 USA

**Keywords:** Drosophila, Transcription levels, TAD, RPKM, Chromosome model, Nuclear envelope

## Abstract

**Background:**

Multiple studies have demonstrated a negative correlation between gene expression and positioning of genes at the nuclear envelope (NE) lined by nuclear lamina, but the exact relationship remains unclear, especially in light of the highly stochastic, transient nature of the gene association with the NE.

**Results:**

In this paper, we ask whether there is a causal, systematic, genome-wide relationship between the expression levels of the groups of genes in topologically associating domains (TADs) of Drosophila nuclei and the probabilities of TADs to be found at the NE. To investigate the nature of this possible relationship, we combine a coarse-grained dynamic model of the entire Drosophila nucleus with genome-wide gene expression data; we analyze the TAD averaged transcription levels of genes against the probabilities of individual TADs to be in contact with the NE in the control and lamins-depleted nuclei. Our findings demonstrate that, within the statistical error margin, the stochastic positioning of *Drosophila melanogaster* TADs at the NE does not, by itself, systematically affect the mean level of gene expression in these TADs, while the expected negative correlation is confirmed. The correlation is weak and disappears completely for TADs not containing lamina-associated domains (LADs) or TADs containing LADs, considered separately. Verifiable hypotheses regarding the underlying mechanism for the presence of the correlation without causality are discussed. These include the possibility that the epigenetic marks and affinity to the NE of a TAD are determined by various non-mutually exclusive mechanisms and remain relatively stable during interphase.

**Conclusions:**

At the level of TADs, the probability of chromatin being in contact with the nuclear envelope has no systematic, causal effect on the transcription level in Drosophila. The conclusion is reached by combining model-derived time-evolution of TAD locations within the nucleus with their experimental gene expression levels.

**Supplementary Information:**

The online version contains supplementary material available at 10.1186/s13072-024-00528-8.

## Introduction

The multi-level organization of the genome in the three-dimensional (3D) space inside the nucleus is believed to be associated with gene expression, but the exact causal connections are far from clear [1, 2]; the importance of 3D chromatin organization for gene regulation was established in some cases [3–7], while in others the effect was weak or non-existent [8–13]. In particular, a possible connection between transcription level of a gene and its position relative to the nuclear periphery has received significant attention and continues to be an active field of research [14–17], see, e.g., these recent reviews [18–23]. A degree of negative correlation has been firmly established between gene expression and its location at the periphery; however, certain aspects of the correlation are still worth investigating, especially in light of newly established properties of chromatin, such as its high mobility even for regions believed to be firmly associated with the nuclear periphery [24, 25]. Critically, the more difficult question of a possible causal connection, the $structure ~ \rightarrow ~function$ causality, still remains open, especially if one moves beyond individual genes, and seeks to establish genome-wide, statistically significant systematic trends. A unique window of opportunity is available here to move forward by combining available experimental data with computational models, which can complement experiments with rich, genome-wide information and resolution (spatial–temporal) otherwise very difficult to obtain purely experimentally. Below is a brief review aimed at justifying these claims that serve as our main motivation.

The nucleus in the eukaryotic cells is separated from the cytoplasm by the nuclear envelope (NE), the internal surface of which is lined by the nuclear lamina (NL)—a meshwork of lamins and associated proteins [18–20]. Lamins, as the major structural proteins of the NL, are considered to be an important determinant of nuclear architecture and gene expression [20]. Multiple early studies have shown a correlation between positioning at the NL/NE and repression of transgenes and individual endogenous genes [26–32]. Some genes that move away from the nuclear periphery, either in lamin mutants (LM) or during tissue differentiation, have been shown to be transcriptionally upregulated [31, 33]. However, expression of other genes appears unaffected by their proximity to the nuclear periphery [29, 34, 35]. The consistency of gene contacts with NL was shown to be negatively correlated with the gene activity (and positively correlated with heterochromatic histone modification H3K9me3) in single cells of the human myeloid leukemia cell line KBM7 [25]. Another study applied scDam&T-seq to reveal how genome–lamina contacts correlate with gene expression in individual cells. A weak genome-wide negative correlation has been found between expression and NL-contacts of genomic regions that infrequently associate with the nuclear periphery [14]. The study indicates that cell-to-cell variations in genome–NE contacts influence gene expression, i.e., regions are more likely to be active in those cells where they are detached from the NE. The authors suggested that the weak association between genome–NL contacts and transcription could be the result of the limited time resolution of these experiments (12 h) and/or the effect of the relatively large 100-kb bins [14]. Genomic regions associated with NL can have highly expressed genes in developing mouse brains, suggesting that NL binding by itself does not repress transcription [36]. Similarly, genome-wide disassociation of genomic regions from NL in *Caenorhabditis elegans* caused upregulation of only a single gene [37].

Several studies directly tested functional consequences of chromatin–lamina association by tethering individual genes to the nuclear periphery in human cells using the lacO/LacI system [38–40]. Since binding of nucleoplasmic LacI molecules to lacO sites in the reporter construct did not, by itself, impair transcription [38, 40], these studies provided direct evidence for a causative role of the nuclear periphery in altering gene expression of many genes. For example, 51 endogenous genes located around a site of induced NE-attachment have been repressed, suggesting that an inactive chromosomal domain has been generated upon tethering to the lamina [40]. Notably, the transcriptional repression caused by the tethering of a gene to the NE was accompanied by histone H4 hypo-acetylation, implying the importance of epigenetic modifications in this process, not necessarily the geometric proximity to the NE *per se*. At the same time, some tethered genes were not repressed [38, 40]. Moreover, other experiments have demonstrated that lamina-targeted genetic loci can still be activated and transcribed at the lamina [39]. The tethering studies suggest that, for some genes, the NL represents a compartment in the cell nucleus that is unfavorable for transcription, but the repression can be overcome by other genes. Overall, these works discovered important features of individual genes and groups of genes, but they did not make genome-wide conclusions.

More recently, chromatin regions called lamina-associated domains (LADs), were systematically identified using a DamID approach in cell nuclei of humans and fruit flies [41–44]. Positioning of LADs relative to the NE appears cooperative: regions with higher linear density of LADs are more likely to be found near the NE [45]. Attachment of LADs to the NL is essentially stochastic: only about 25–30% of all LADs in the fruit fly are located at the nuclear periphery at any given moment in any given cell [41, 43, 46]. Similar to other chromosome loci in the interphase nuclei [47–51], the fruit fly LADs are highly mobile within each nucleus. Despite having a relatively strong affinity to the NL [45], most fruit fly LADs come in contact with the NL and move away from it multiple times during the interphase. Therefore, tethering experiments in which a locus is permanently anchored to the NL, or DamID studies with a limited temporal resolution of NL-contacts during the interphase, may not represent the highly mobile nature of chromatin in fruit flies. Forced expression of several genes located mostly at the nuclear periphery can cause their detachment from the NL [52]. Genome-wide gene activation inside LADs typically causes detachment of LADs from the NL, but it usually does not involve more than 50 kilobases of DNA flanking the activated gene, and the NL detachment is dependent on transcription elongation [53].

An experiment that can probe, directly, whether a statistically meaningful change in the necessarily stochastic position of a gene relative to the NE causes a systematic, statistically meaningful change in the gene expression (the $structure ~ \rightarrow ~function$ causality) would be most appropriate, but to the best of our knowledge, no such studies have been performed on a genome-wide scale. For example, a recent pioneering genome-wide analysis of gene transcriptions in control vs. Lamin-knockdown (Lam-KD) fruit fly nuclei [46] demonstrated that, upon Lam-KD, a number of genes in LADs increased their transcription levels by up to a factor of 4, while for another subset of genes, a decrease by up to a factor of 2 was observed. However, changes in the cumulative spatial distributions of the chromatin regions upon Lam-KD were revealed for only three genomic regions in LAD-containing TADs, which speaks for the difficulty of performing such experiments for every gene.

The difficulties in performing this kind of analysis purely experimentally motivate combining experiment with an appropriate computer modeling [45, 54, 55] to make progress. Here, an experiment can provide gene expression data, while computer modeling can trace the movement of the relevant chromatin units with the desired spatial and temporal resolution on a genome-wide scale to detect systematic trends if any.

Due to the highly stochastic nature of transcription at the level of individual genes, relatively large units of chromatin structure—beyond an individual gene—are more appropriate for the goal of exploring systematic trends in gene expression by averaging over many genes that are close to each other in 3D space. Our specific choice is introduced below.

Application of the chromosome conformation capture technique with high-throughput sequencing (Hi-C) to study chromatin organization identified topologically associating domains (TADs) in various organisms of high eukaryotes [56–60]. TADs may contain multiple genes, range in size from tens of kilobases (kb) in fruit flies to several megabases (Mb) in mammals, and represent structural and functional units of three-dimensional (3D) chromatin organization [61–64]. Since the boundaries between TADs are conserved among cell types and sometimes across species [57, 65–72], a TAD can be regarded as a natural unit of genome partitioning for exploring systematic structure–function relationships in chromatin. Certain TADs overlap with LADs in cell nuclei of humans and fruit flies [42–44, 57–59].

In this work, we combine published [46] genome-wide gene transcription data with a novel modeling approach to ask whether there is a causal, systematic relationship between the transcription level of the groups of genes in TADs of *Drosophila* nuclei and the probability of TADs to be found at the NE, taking into full account the highly mobile nature of the TADs. For this purpose, we employ a recently developed model of the fruit fly interphase chromatin [45] that describes the dynamics and spatial organization of the entire diploid set of female chromosomes at TAD resolution, and their interactions with the NE. The model, which accounts for different epigenetic classes of TADs, TAD–TAD contact probabilities (Hi–C map), and the known distribution of LADs along the genome, reproduces experimentally observed chromatin density profiles for both control and lamins-depleted nuclei and can faithfully predict the probabilities of individual TADs to be in the layer adjacent to the NE. To investigate the genome-wide relationship between the positioning of TADs at the NE and gene transcription at TAD resolution, a robust TAD-averaged metric of transcription activity is introduced.

## Results

### TAD transcription levels and the probability of a TAD to be in contact with the NE: correlation

We begin by investigating whether average gene transcription levels in TADs, which are relatively free to move within the fruit fly interphase nuclei [45, 47, 51, 73], correlate with average TADs geometric proximity to the NE. While we do expect such a correlation, in general [74], we aim to investigate it in detail; for example, it may exist only for a certain subgroup of TADs. As the next step, we would like to test whether the positioning of TADs at the NE *per se* causes the change in transcription.

To this end, we need a probabilistic metric for the TAD–NE proximity, because the same TAD can move in and out of proximity to the NE multiple times during the interphase [45]. It is reasonable to assume that, if proximity to the NE suppresses transcription, the degree of suppression of gene transcription activities in a TAD should be related to the fraction of time the TAD spends in contact with the NE.

This fraction can be expressed as the probability of a TAD to be in a narrow contact layer near the NE, see Materials and Methods and Figure S1 in Additional file 1. We consider this probability (frequency) of a TAD to be in contact with the NE as a suitable measure of the TAD’s *stochastic* proximity to the NE.

In this work, we introduce and employ a normalized metric for gene transcription levels at TAD resolution, $\textrm{RPKMT}$. This metric extends the commonly used single gene transcription level metric, RPKM (reads per kilobase of transcript per million reads mapped), to TAD resolution reflecting a TAD averaged transcription activity, see Materials and Methods. To make sure that our main conclusions are robust to details of the transcription metric definition, we also consider another metric of transcription levels in TADs, $\textrm{RPKMTL}$ (number of reads mapped to all genes in a TAD per kilobase of TAD length per million reads mapped to all TADs), see Additional file 1. In $\textrm{RPKMTL}$, a TAD length instead of a sum of gene lengths in a TAD (in $\textrm{RPKMT}$) is used.

**Fig. 1:**
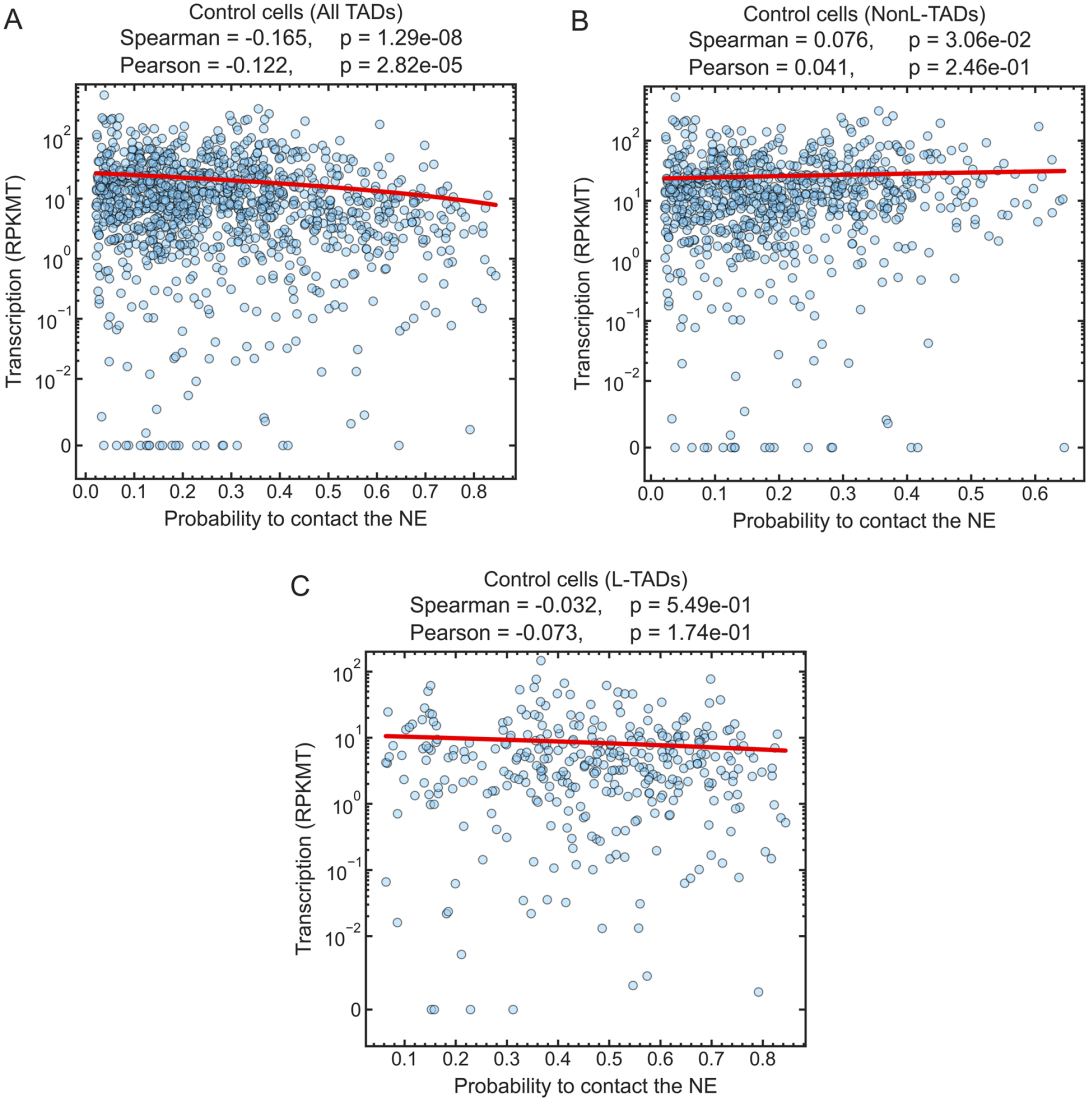
Transcription levels, in $\textrm{RPKMT}$, of genes in each TAD vs. the probability of a TAD to be in contact with the NE in the control cells. **A** A weak negative correlation is seen when all TADs are considered. **B**, **C** The scatter plots show essentially no correlation for NonL-TADs or L-TADs, considered separately. The Spearman and Pearson correlation coefficients, their two-sided *p* values (*p*), and linear regression lines (red) are shown

Figure 1A shows that there is a weak negative correlation between the transcription activity in TADs and their probability to be in contact with the NE in the control (i.e., with the intact lamina) *Drosophila* cells. The absence of a pronounced negative correlation may be unexpected, as the transcription of genes in LADs is well known to be repressed [20, 40, 75], and, therefore, it is also expected to be repressed in TADs containing LADs (L-TADs), which interact with the NL and constitute a significant (about 30%) fraction of TADs in *Drosophila* [43, 59]. Nuclear periphery is generally assumed to be an inactive compartment [51].

To gain further insight into the weak correlation seen in the control cells nuclei (Fig. 1A), we separate the TADs into two major groups: TADs not containing LADs (NonL-TADs) and TADs containing LADs (L-TADs), see Fig. 1B and C. We see essentially no correlation between the transcription levels ($\textrm{RPKMT}$) in L-TADs/NonL-TADs and the probability of L-TADs/NonL-TADs being in contact with the NE in control cells. The significantly lower average gene transcription levels in L-TADs relative to NonL-TADs (compare the levels of linear regression lines in Figs. 1B, C) and the absence of NonL-TADs with the probabilities to be in contact with the NE greater than 0.65 explain the weak negative correlation between the transcription levels and the NE contact probabilities for all TADs combined, as seen in Fig. 1A.

**Fig. 2:**
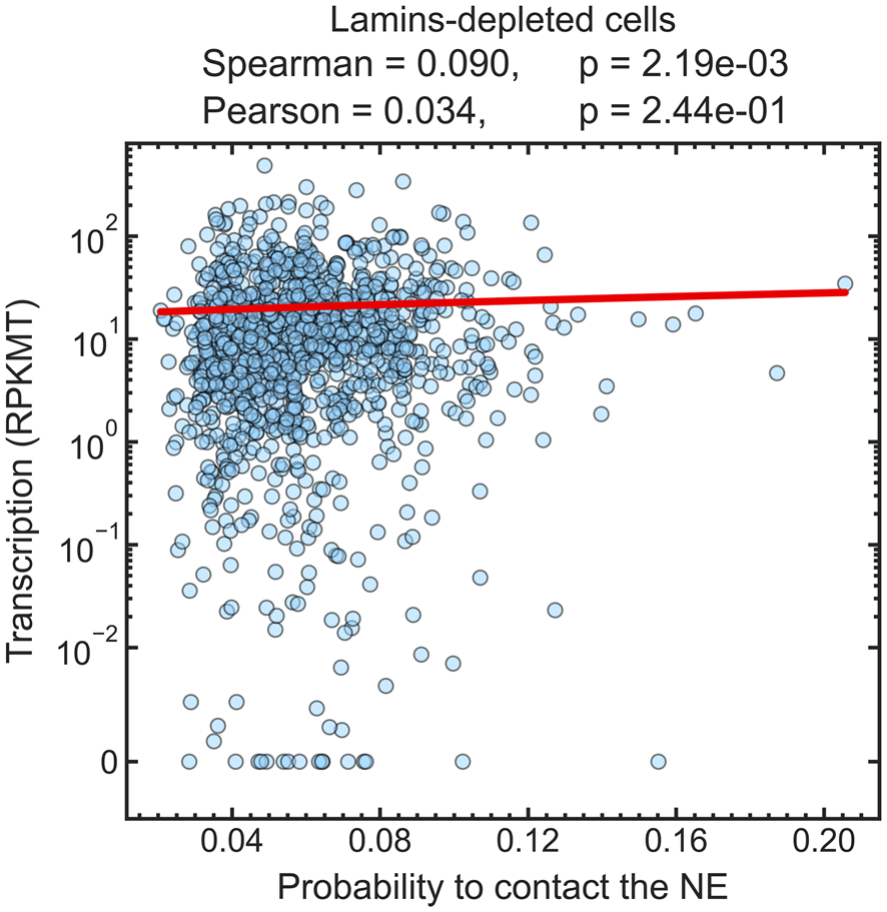
Essentially no correlation is seen between the transcription levels in all TADs (in $\textrm{RPKMT}$) in the lamins-depleted cells and the probability of TADs being in contact with the NE. The Spearman and Pearson correlation coefficients, their two-sided *p* values (*p*), and the linear regression line (red) are shown

Given the already weak correlation between the transcription levels of TADs and the probabilities of TADs to be in contact with the NE, seen in the control nuclei, it is not surprising that no correlation between the transcription activities in TADs and their contact probabilities is seen in the lamins-depleted nuclei, where fewer TADs are expected to be in contact with the NE relative to the control cells, Fig. 2. In what follows, we investigate whether a weak signal is still there, “drowned out” by the inherently noisy data.

To discern meaningful, systematic, genome-wide trends behind the large natural variation of transcription activity between individual TADs, as well as inevitable errors in the experimentally reported gene transcription levels, we propose averaging transcription levels even beyond individual TADs, which will help us to focus on systematic trends. We also argue for comparisons of mean values directly, rather than of means of ratios [46], as the latter can contain unintended biases towards higher values due to inevitable uncertainties, see the discussion next to Table S1 in Additional file 1.

**Fig. 3:**
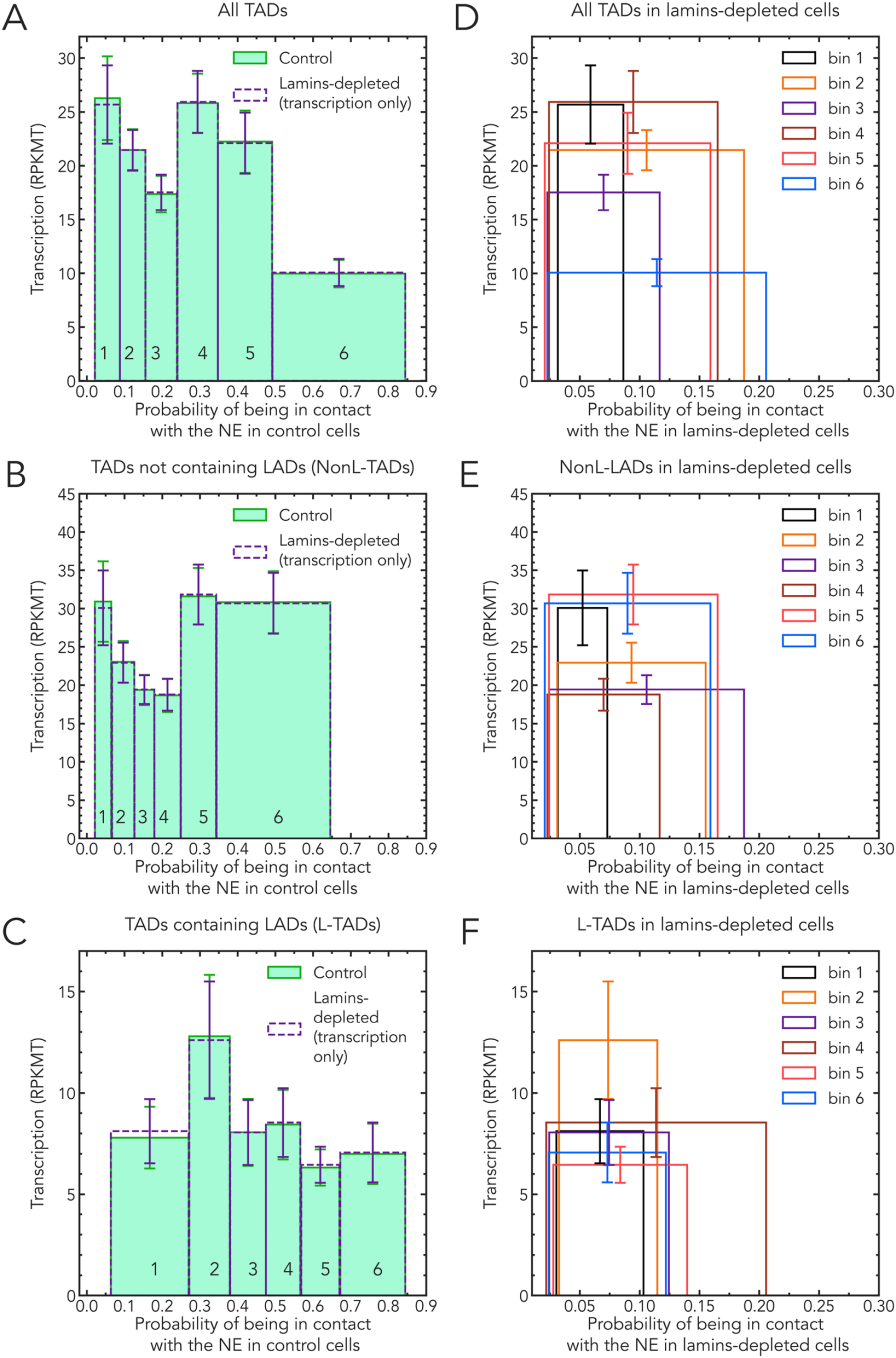
Dependencies of bin averaged TAD transcription levels (in $\textrm{RPKMT}$) on the probability of TADs in the bin to be in contact with the NE. Virtually no change in the transcription levels—bin heights—is seen in lamins depleted cells compared to control. The binning of TADs is based on TAD-NE contact probabilities in control cells for each set (selection) of TADs. Solid bars: control cells. Empty bars: lamins-depleted cells. The same set of TADs per bin is used in the control and lamins-depleted cells. Error bars are s.e.m. (standard error of the mean). Left panels: **A** all TADs; **B** TADs not containing LADs (NonL-TADs); and **C** TADs containing LADs (L-TADs). In the left panels only, the positions of the empty bins (lamins-depleted cells) along the x-axis are deliberately kept unchanged to facilitate visual comparison of the bin heights between lamins depleted and control bins. Right panels show only lamins-depleted cells: **D** all TADs; **E** TADs not containing LADs (NonL-TADs); and **F** TADs containing LADs (L-TADs). A clear shift of the average TAD positions away from the NE is evident

To this end, we have binned the TADs (separately all TADs, L-TADs and NonL-TADs) based on the probability of a TAD in control cells to be in contact with the NE, see Materials and Methods. Each of the six bins in Fig. 3A (B,C) contains an approximately equal number of TADs, see Materials and Methods. The heights of the bars indicate the average transcription level of TADs (in $\textrm{RPKMT}$) in each bin. The binning procedure allows us to compare changes in transcription activity of groups of TADs that differ by the probability to be in contact with the NE.

Considering all TADs (i.e., both NonL-TADs and L-TADs, Fig. 3A), the TADs in bin #6—TADs that are most likely to be in contact with the NE—do show a marked (2x) decrease in transcription levels compared to TADs in other bins with a lower probability of being in contact with the NE. This trend, however, does not continue for TADs in bin #5, with the next highest level of contact probabilities. The average transcription level in bin #5 is roughly the same as those of bin #1 and #2 that contain TADs with the very low (less than 0.15) contact probabilities.

**Fig. 4:**
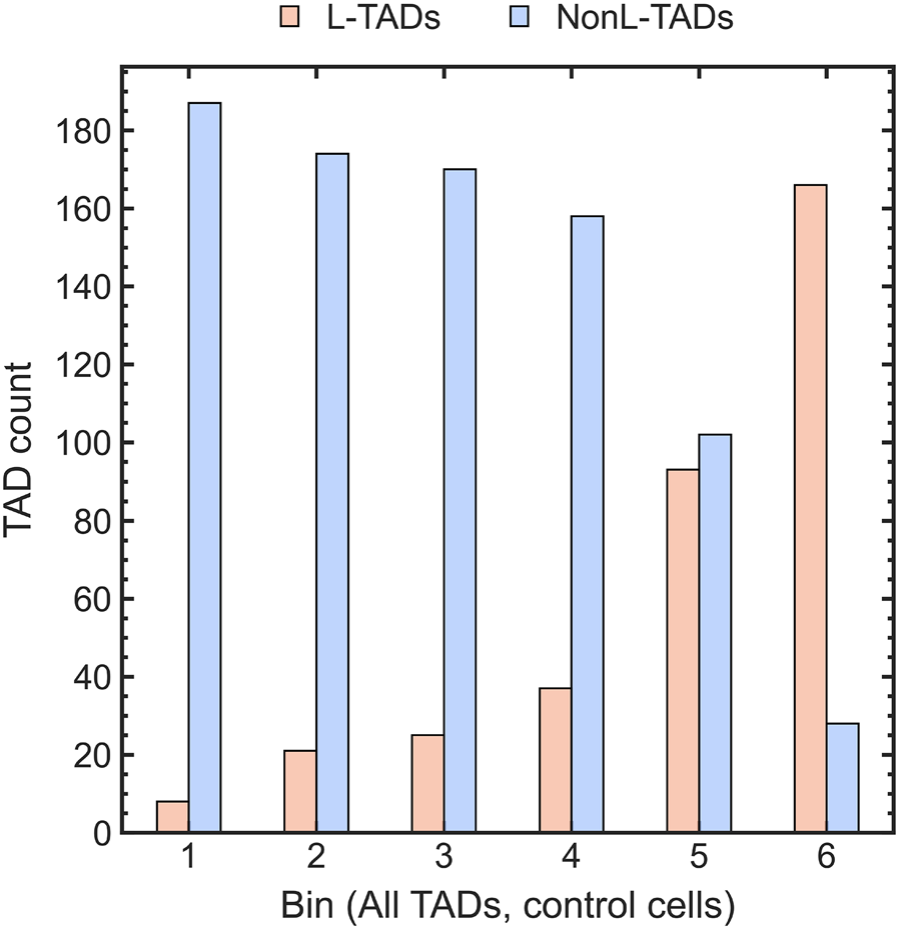
Comparison of the number of L-TADs and NonL-TADs in each bin of control cells for All TADs. The number of L-TADs in each bin increases with the stochastic proximity to the nuclear envelope (the probability of a TAD to be found at the NE increases from bin # 1 to bin # 6)

As we suggested earlier, analyzing data in Fig. 1, the abrupt drop in the transcription seen in bin #6 is due to a very high fraction of L-TADs in this bin (see Fig. 4). These LAD-containing TADs have 2 to 4 times lower average TAD transcription levels compared to NonL-TADs (see Fig. 3B and C, and Ref. [46]). Although a small fraction of genes located in LADs can actively express (about 10%), transcription of the majority of genes inside LADs is repressed to a very low level [41–43, 46, 76, 77], leading to relatively low levels of transcription in L-TADs. No correlation of the average transcription levels with the likelihood of being in contact with the NE is seen for NonL-TADs in Fig. 3B. The drops in the transcription levels seen in bins #3 and #4 (Fig. 3B) are tangential to the main focus of this work, and so we do not pursue their origins here; a relevant hypothesis is outlined in Additional file 1, Figure S7.

In summary, our first conclusion is that the weak negative correlation between the transcription level of all TADs and their stochastic proximity to the NE in the control cells is due to an exceptionally large fraction of L-TADs among TADs with the highest probabilities (greater than 0.5) to be in contact with the NE and much lower (3–4 times) average transcription levels in L-TADs compared to NonL-TADs.

### TAD transcription levels vs. the probability of a TAD to be in contact with the NE: no causation.

A natural question arises whether the weak negative correlation between the transcription activity of TADs and their stochastic proximity to the NE in fruit fly nuclei is causal. In other words, does the increased probability of finding a TAD in contact with the NE, by itself, *cause * the transcription level to decrease?

To address this question, we have considered the transcription patterns in lamins-depleted cells. First, note that the lamins knockdown or a mutation causes a drastic spatial re-arrangement of chromatin within the nucleus [45, 46, 78]—most of the TADs move away from the NE, see Figs. 3D,E,F, as well Figure S2 in Additional file 1. For example, LamA25 mutation [78] removes the CaaX motif from Lamin B and disrupts its attachment to the nuclear membrane, thus, depleting Lamin B from the nuclear periphery [78, 79]. In fact, the probability of finding most of the TADs in contact with the NE in lamins-depleted nuclei is less than 0.16, with no bins extending beyond 0.21, Figs. 3D–F. See also Figure S1 in Additional file 1 for the stark contrast between the control and lamins-depleted cells in this respect. If being in contact with the NE with an appreciable probability caused a *systematic* drop in the transcription of TADs containing LADs, the average transcriptional activity of these TADs would be expected to recover almost fully in lamins-depleted nuclei. Yet, virtually no change in average transcription levels is seen in each of the *same* groups of TADs (bins), Fig. 3, open bars (lamins-depleted) vs. solid bars (control). Note that we deliberately kept exactly the same sets of TADs in each lamins-depleted bins as in the control bins.

Thus, our second and main conclusion is that stochastic proximity to the NE of TADs, which are highly mobile, does not, by itself, cause a noticeable *systematic, genome-wide* change in transcription at TAD resolution. The key conclusions mentioned above are robust with respect to the metric of transcription activity used, see Figures S3, S4 in Additional file 1, where an alternative metric was employed.

## Discussion

This work has combined simulation and experiments to arrive at the following main result: the probability of a TAD to be found in contact with the NE, by itself, has no systematic, causal effect on its transcription level in *Drosophila*. We stress several key aspects of this statement. First, it applies to a systematic trend only, averaged across multiple TADs, and not necessarily to experiments in which individual TADs are perturbed, e.g., permanently tethered to the NE. As shown in [46], it is entirely possible for transcription levels of a subset of individual, weakly expressing TADs to increase in lamins-depleted nuclei, where their probability to be in contact with the NE is expected to decrease relative to the control nuclei. There is no contradiction here with our main claim: even if more TADs become transcriptionally up-regulated then down-regulated upon lamins depletion, which was indeed seen in Ref. [46], the net effect on transcription, averaged over large groups of TADs, can still be zero. And this is what we are finding, within the statistical margin of error. In addition, lamins knockdown used in [46] can affect gene expression in more than one way, including an increase of the acetylation level at histone H3 and a decrease of chromatin compaction in LADs.

Second, we stress the highly mobile nature of TADs [45], including L-TADs, reflected in our deliberate choice of words in “probability to be in contact with the NE” *vs.* the more common “proximity to the NE”. We have also used “stochastic proximity” to emphasize the same point. L-TADs, even those that are likely to be found in contact with the NE, move away from the NE multiple times during the interphase [45]. Thus, the condition of a stable, essentially permanent attachment to the NE, *e.g., via* a tether, is not reproduced in live *Drosophila* nuclei—this observation removes the perceived contradiction with the pioneering tethering studies that demonstrated down-regulation of certain loci upon induced contact with the NE [38–40]. Indeed, targeting of lamin-associated loci does not take place during interphase when loci can only form transient contacts with the lamina [39]. Stable tethering of a locus to the nuclear lamina requires passage through mitosis. Similarly, treatment of *Drosophila* S2 cells with dsRNA against lamin Dm0 was performed over four days [46] allowing passages through mitosis. It is likely that cell division is necessary for the re-setting of an epigenetic state of the chromatin at the nuclear periphery. During mitosis most modifiers of histone tails are removed from chromatin, and higher order architecture is lost to facilitate proper chromosome condensation and segregation [80, 81]. Epigenetic marks are accumulated mainly during the G1 phase and remain relatively stable during the same interphase [82, 83], despite the fact that TADs frequently change their positions with respect to the lamina.

Different mechanisms for LAD positioning and repression are not expected to be mutually exclusive. For example, a study performed a detailed analysis of gene repression mechanisms in LADs using human K562 cells [84]. By systematically moving promoters from their native LAD location to a more neutral chromatin environment and by transplanting them to a wide range of chromatin contexts inside LADs, the study has demonstrated that the variation in the expression level can be due to the interplay between the promoter sequence and local chromatin features in the LADs [84]. Chromatin features in the LADs can be partially responsible for interaction with the lamina, since lowering H3K9me2/3 levels decreases LAD-contacts in human cells [24, 25]. Thus, the lamina can attract loci of inactive chromatin contributing to the assembly of repressive peripheral chromatin domains. The NE proteins can also attract chromatin to the lamina and participate in the repression of transcription. For example, histone deacetylase HDAC3, which is associated with the NE transmembrane (NET) protein Lap2beta and the DNA-binding protein cKrox, was shown to attract LADs to the nuclear periphery in mouse cells [85, 86] and Drosophila S2 cells [87]. Inhibition of histone deacetylation with trichostatin A (TSA) in *Drosophila* cells causes loss of Lamin binding to chromatin [41]. Thus, nuclear lamina-associated HDACs could contribute to the repressive environment of the nuclear periphery by downregulating gene expression [88, 89] and assembling the peripheral chromatin. Alternatively, the radial segregation of chromosomes based on gene density has been linked [90] to transcription that segregates the active chromatin associated with the nuclear interior from the inactive chromatin located more peripherally. Moreover, lamins themselves can affect both the position and transcription of chromatin. A study has shown that artificial anchoring of lamins to promoters of transfected reporter plasmids can lead to reduced transcription [91]. A conditional and temporal up-regulation of lamin C in *Drosophila* caused a shift in chromatin distribution from peripheral to central, which was associated with reduced levels of the active chromatin mark H3K9ac [92]. Depletion of lamin Dm0 resulted in the moderate upregulation of the generally very weak background transcription in LADs but not in the inter-LADs in *Drosophila* S2 cells [46]. Also, lamin B1 depletion decreased heterochromatin marker H3K27me3 by 80 percent in human cells [93]. While confirming the well-known repressive role of the NE, we make a conclusion here that the expected (weak) negative correlation between the TAD transcription level and its stochastic proximity to the NE disappears completely for TADs containing lamina-associated domains (L-TADs) or not containing them (NonL-TADs), considered separately. The presence of the correlation for all TADs combined is explained by the significantly lower average gene transcription levels in L-TADs relative to NonL-TADs and the absence of NonL-TADs with high probabilities to be in contact with the NE. Despite certain differences in nuclear protein composition and functions, the key organizational principles of TADs, LADs, chromatin and nuclear compartments are similar among different organisms [94–98]. The aforementioned studies suggest that our general conclusions may apply to both insects and mammals.

**Fig. 5:**
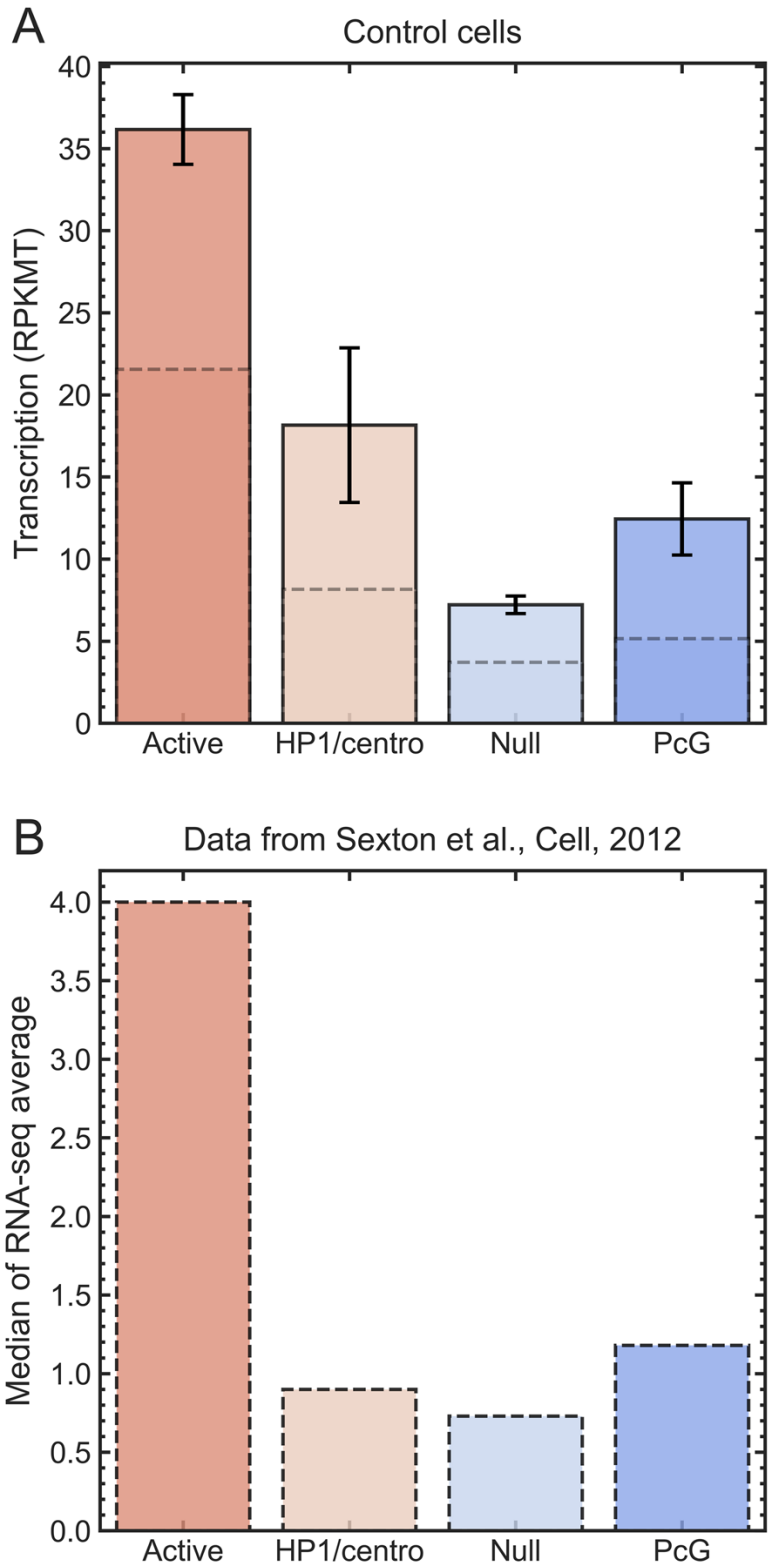
The proposed metric for transcription activity in TADs, $\textrm{RPKMT}$, is consistent with the transcription activities in different epigenetic classes of TADs identified previously by Sexton et al. [59]. Panel **A:** Mean (solid boxes) and median (dashed lines) transcription levels (in $\textrm{RPKMT}$) for Active TADs (n=494), HP1/centromeric (n=52), Null (n=492), and PcG (n=131) epigenetic TAD classes. Error bars are standard errors of the mean. Both mean and median $\textrm{RPKMT}$ transcription levels in Active TADs are at least 2 times greater than those in the three repressive epigenetic TAD classes (Null, PcG, and HP1/centromeric). These $\textrm{RPKMT}$ transcription levels demonstrate consistency with the gene transcription levels (in medians of RNA-seq averages) within each epigenetic class of TADs shown in Figure 3C of Ref. [59], reproduced in Panel **B**

Our study has several limitations. The conclusions rely on predictions of a computational model: while the systematic, and even a few individual trends in the predicted TAD positioning have been verified against experiment, deviations from reality may still occur on the level of individual TADs. Thus, we stress the systematic nature of the main conclusion, likely correct in the average sense. A related limitation is that the minimal structural unit of chromatin employed in this work is a TAD, which is about 100 kb for *Drosophila*. We do not make any claims about what might be happening at finer resolutions, including promoter–enhancer interactions within TADs. We stress that the resolution limitation does not invalidate our main conclusions based on averages. For example, it is known that individual active genes within a LAD can locally detach from the lamina. But still, genes belonging to a LAD that is stochastically closer to the NE are, on average, closer to the NE than genes in a LAD that is stochastically further away from the NE. Therefore, conclusions that rely on averages over these genes remain valid. Another possible limitation is that the cell types used to define the epigenetic classes employed by the computational model and to generate the transcription profiles (RNA-seq) were taken from related, but not exactly the same cell cultures. The concern is mitigated by the fact that both cell types have an embryonic origin. Moreover, we have confirmed the consistency of the transcription profiles within the four epigenetic classes of TADs between S2 embryonic cell line data [46] used in our study, Fig. 5A, and 16–18 h embryos data [59] presented in Fig. 5B. The data from this latter work ( [59]) were used to develop parameters of the computer model [45] from which we compute the TAD–NE contact probabilities. The fourth limitation is that the lamin knockdown may not only cause global chromatin relocation but also epigenetic perturbation of TADs. This concern is mitigated by the fact that the level of histone H3 acetylation is elevated only in LADs, but not in the LAD-free regions upon Lamin knockdown when compared to control cells [46]. Thus, the epigenetic profiles of the majority of TADs are expected to be unchanged upon the Lamin knockdown.

### Notes on possible mechanisms and testable hypotheses

The main implication of this work is that, with some relatively rare exceptions, the role of the NE does not include significant *systematic* regulation of transcription states of genes in TADs. Instead, the lamina may help to “lock in” the repression state of L-TADs by more than one mechanism. First, the nuclear lamina may facilitate better separation of active and inactive chromatin by sequestering inactive TADs (Null, PcG, and HP1/centromere) at the nuclear periphery. This can be achieved by yet unknown mechanisms of attraction between the NE proteins and specific histone modification and/or chromatin proteins. In this mechanism, the epigenetic profile of TADs determines both their affinity to the NE and transcriptional repression. That would explain both the presence of the negative correlation of transcription levels of L-TADs with the probability of being in contact with the NE and the absence of any causal connection to transcription. In this picture, we assume that the state of being in affinity to the NE is set outside the interphase, at least outside the G1 phase that we model. Second, the NE may contain proteins that interact with gene-poor chromatin and define the epigenetic status of TADs by modifying their histone tails. In this mechanism, the initial postmitotic interaction of a chromatin locus with the NE proteins sets its “LAD status” for the rest of the interphase. These settings include the accumulation of repressive epigenetic marks and frequent contact with the NE during the interphase. Experimental support exists for both of these mechanisms, see Discussion. In summary, we put forward two non-mutually exclusive hypotheses for future testing.

*Scenario 1: * NE–chromatin contacts and transcription are controlled by the same mechanism. The epigenetic profile of a LAD is set by chromatin proteins or other nuclear proteins at the onset of the interphase, and it determines both affinity to the NE and transcriptional repression. In this scenario, epigenetic repression of transcription leads to affinity for the NE.

*Scenario 2:* NE–chromatin contacts and transcription are controlled by different mechanisms. The epigenetic profile of a LAD is set by NE proteins during the initial LAD–NE interaction after mitosis and it determines persistent transcriptional repression for the rest of the interphase. In this scenario, affinity of LADs for the NE leads to epigenetic repression of transcription.

A way to reconcile our general findings with the tethering experiments described in the introduction is to assume that the epigenetic state of a TAD can be reset by contact with the lamina, but that reset requires a cell going through mitosis and forming a new NE, after which the locus has a relatively long and persistent affinity to the NE. Likewise, once the epigenetic state is set, a long and persistent absence of contact is needed for a return to the original state. Only a subset of TADs is liable to such a reset. The underlying assumption is that a relatively long time is needed to reset the epigenetic state, as appears to be the case in experiments where the epigenetic state is reset by mechanical compression of the nucleus [99].

## Materials and methods

### Normalized measure of gene transcription levels at TAD resolution

RNA-seq methods generate data that needs to be normalized to eliminate technical biases associated with the methods, such as the sequencing depth of a sample and the length of the mRNA transcripts [100]. To correct these biases, RPKM (reads per kilobase of transcript per million of total mapped reads) measure [101] has been widely used:

1 $$\begin{aligned} \textrm{RPKM}=\dfrac{ 10^6 \times \text{ Reads } \text{ mapped } \text{ to } \text{ transcript } }{\text{ Total } \text{ mapped } \text{ reads } \times \text{ Transcript } \text{ length } \text{ in } \text{ kb }} \end{aligned}$$

To quantify the average gene expression levels in a TAD, we propose a similar measure—$\textrm{RPKMT}$ (number of reads mapped to all genes in a TAD per kilobase of the total length of mapped genes in a TAD per million of total mapped reads). This metric normalizes for the sum of all gene lengths in a TAD and the sequencing depth of the sample. $\textrm{RPKMT}$ characterizes an average expression of all genes in a TAD, and is defined as

2 $$\begin{aligned} \textrm{RPKMT}= \dfrac{ 10^6 \times \text{ Reads } \text{ mapped } \text{ to } \text{ genes } \text{ in } \text{ a } \text{ TAD } }{\text{ Total } \text{ mapped } \text{ reads } \times \text{ Total } \text{ length } \text{ of } \text{ mapped } \text{ genes } \text{ in } \text{ a } \text{ TAD } \text{ in } \text{ kb }} \end{aligned}$$

### Calculation of $\textrm{RPKMT}$ from published RNA-seq data

The RNA-seq data used here contains two replicates (rep1 and rep2) of control S2 cells (samples GSM3449348 and GSM3449349) or lamins-depleted cells (samples GSM3449350 and GSM3449351) [46]. The TADs and their genomic coordinates are defined in [59]. For chromosome arms 2L, 2R, 3L, 3R, 4 and X, the FlyBase IDs of the genes (FBgn#), the genomic coordinates of the genes on the chromosomes are extracted from the following FASTA files: https://ftp.flybase.net/releases/FB2008_09/dmel_r5.12/fasta/dmel-2L-gene-r5.12.fasta.gz, https://ftp.flybase.net/releases/FB2008_09/dmel_r5.12/fasta/dmel-2R-gene-r5.12.fasta.gz, https://ftp.flybase.net/releases/FB2008_09/dmel_r5.12/fasta/dmel-3L-gene-r5.12.fasta.gz, https://ftp.flybase.net/releases/FB2008_09/dmel_r5.12/fasta/dmel-3R-gene-r5.12.fasta.gz, https://ftp.flybase.net/releases/FB2008_09/dmel_r5.12/fasta/dmel-X-gene-r5.12.fasta.gz. The genomic lengths of the genes (label “length”) are extracted from the FASTA file: https://ftp.flybase.net/releases/FB2008_09/dmel_r5.12/fasta/dmel-all-gene-r5.12.fasta.gz. A gene is assigned to a TAD if: (i) the genomic start coordinate of the gene is located within the TAD, or (ii) the genomic start coordinate of the gene is less than or equal to the genomic end coordinate of the TAD and the genomic end coordinate of the gene is greater than or equal to the genomic end coordinate of the TAD, according to BDGP Release 5.12/dm3. A gene is assigned to one TAD only. The mapped genes are the genes that are assigned to TADs and are found in the replicates. As a result, there are 8 out of 1169 TADs that do not contain any genes. Therefore, in this work, we deliberately set numbers of reads in these TADs to zero. The scripts and data sets used in the study are available at: https://github.com/Onufriev-Lab/NE_TRANSCRIPTION/.

Taking the two replicates into account, we calculate the transcription activity metric ($\textrm{RPKMT}$), initially defined by Eq. 2, as

3 $$\begin{aligned} \textrm{RPKMT}= \dfrac{ 10^6 \times (\text{ Sum } \text{ of } \text{ reads } \text{ of } \text{ rep1 } \text{ and } \text{ rep2 } \text{ mapped } \text{ to } \text{ genes } \text{ in } \text{ a } \text{ TAD})}{\text{(Total } \text{ mapped } \text{ reads } \text{ of } \text{ rep1 } \text{ and } \text{ rep2) } \times \text{ Total } \text{ length } \text{ of } \text{ mapped } \text{ genes } \text{ in } \text{ a } \text{ TAD } \text{ in } \text{ kb }} \end{aligned}$$

We have verified that the proposed metric of transcription activity, Eq. 3, used with the RNA-seq data from Ref. [46], is consistent with the transcription activities in different epigenetic classes of TADs [59], which are employed by our computational model. The consistency is demonstrated in Fig. 5 (see also Figure S5 in Additional file 1). Specifically, we consider two transcription profiles to be consistent if their corresponding medians over TAD classes satisfy the following criterion: the transcription level of Active TADs is much higher than those of all other epigenetic TAD classes. This consistency check mitigates potential concerns related to inevitable, but minor, differences in gene expression profiles that may stem from differences between the types of embryonic cells from Ref. [46] (S2 embryonic cell line) vs. those [59] (embryos collected 16–18 hrs after egg laying) employed to build the dynamic computational model used here. Another possible source of the observed differences in gene expression profiles shown in Fig. 5, specifically a lower ratio of Active to non-Active median TAD levels in our $\textrm{RPKMT}$-based transcription profile (Fig. 5A) compared to the profile from Ref. [59] (Fig. 5B), may be related to the fact that, by construction, $\textrm{RPKMT}$ metric normalizes for both the coding and non-coding regions of the genes that are located in a TAD. Moreover, due to the overlapping of the genomic regions of genes in a TAD and the way the genes are assigned to a TAD (i.e., by their genomic start coordinate), the sum of the gene lengths in a TAD sometimes can exceed the length of a TAD.

While the proposed TAD-based metric of gene transcription might be applicable outside this work, we stress that no claims of its general applicability are made. In particular, we have not investigated to what extent $\textrm{RPKMT}$ can be used to compare transcription levels between different cell types or organisms, which would be out of scope here. All that we require here is that $\textrm{RPKMT}$ is a monotonic function of transcription activity, *i.e.,* if the latter is decreased as a result of a gene knockdown, the value of $\textrm{RPKMT}$ reflects that.

### The dynamic model of interphase chromosomes at TAD resolution

To determine the probabilities of TADs to be in contact with the NE, we use a dynamic model of a *D.melanogaster* female interphase nucleus with a diploid set of four homologous chromosomes, developed in our previous work [45]. A brief description of the model is given below.

**Fig. 6:**
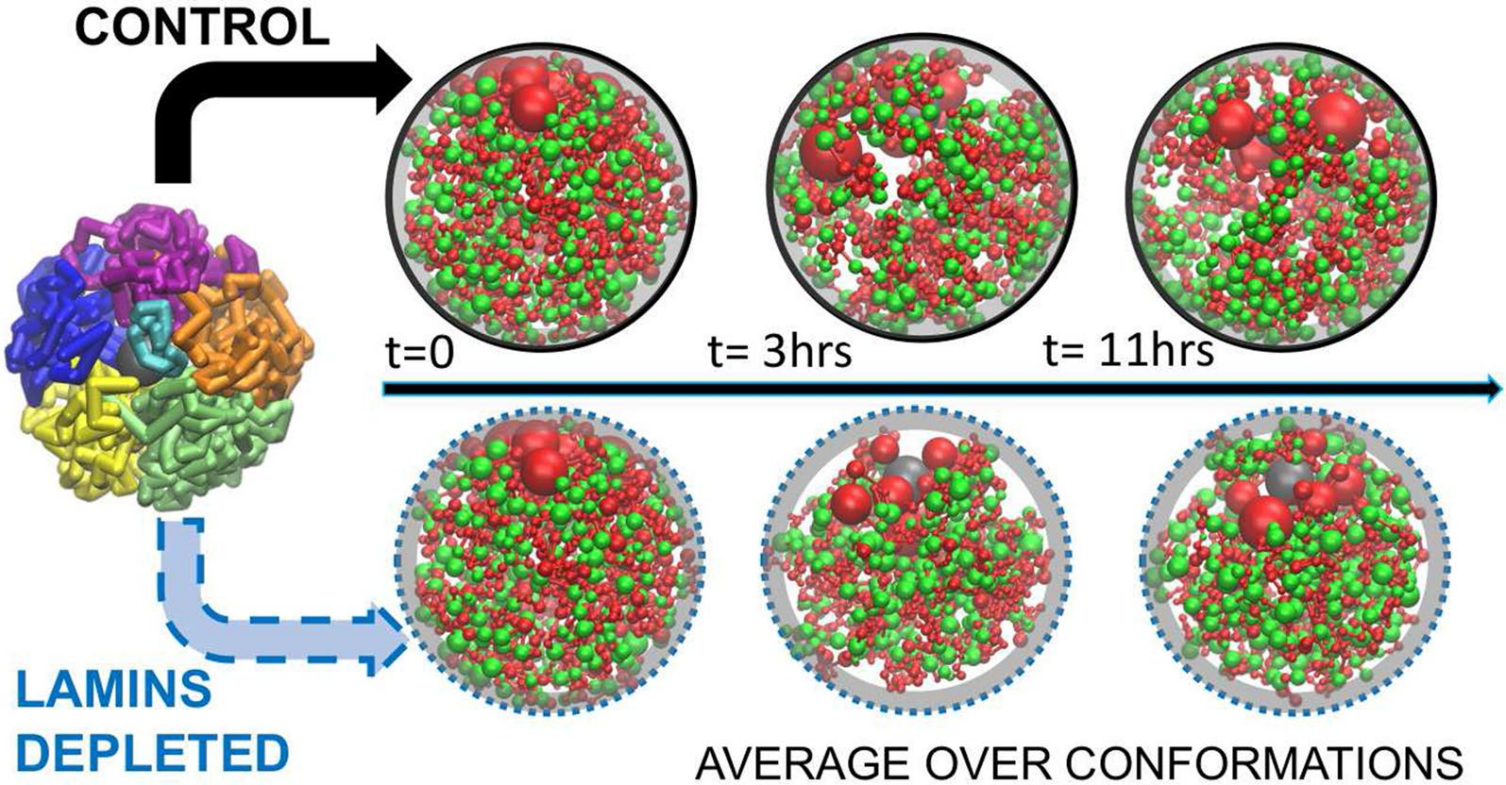
The dynamic model of fruit fly nucleus at TAD resolution. Starting from an initial arrangement of the chromosome arms (leftmost), the chromatin in control (upper row) and lamins depleted (bottom row) nuclei changes conformation as time progresses along the interphase. Shown are actual snapshots from the respective simulations and their corresponding approximate biological time points [45]. See supplementary movies of the chromosome arms motion: Additional files 2, 3, 4. Structural signatures, such as probability of a TAD to be in contact with the NE, are computed and averaged along each trajectory. Green spheres: L-TADs. Red spheres: NonL-TADs. The grey spherical shell next to the NE indicates the 0.2 $\mu$m contact zone

The model simulates dynamics of the chromatin fibers in both control and lamins-depleted nuclei for a time equivalent to 11 hrs (the duration of the *D.melanogaster* nucleus interphase) and allows to analyze the trajectories of individual TADs in both control and lamins-depleted nuclei, Fig. 6. Langevin dynamics simulations have been performed using ESPResSo 3.3.1 package [102] by solving the Langevin equations of motion.

In the model, each pair of homologous chromosomes (2, 3, 4 and X), which are in proximity to each other [103], is represented by a single chain of spherical beads using "beads-on-a-string" model [104, 105]. These chains of beads are surrounded by a spherical boundary representing the NE. For biological realism, all of the experimentally observed distinct mutual arrangements (topologies) of *D.melanogaster* chromosome arms [106] are considered and simulated [45]. One of these arrangements is presented in Fig. 6, leftmost panel. Each of the 1169 beads (red and green spheres in Fig. 6) in the chromosome chains corresponds to one of 1169 pairs of homologous TADs—physical domains resolved in the Hi–C maps [59]. In addition, 4 beads represent centromeric chromatin domains (CEN) in each chain, and 6 beads adjacent to CEN beads represent pericentromeric constitutive heterochromatin (HET) domains (larger red spheres in Fig. 6). The mass and the size of each bead correspond to the length of the DNA contained in the corresponding TAD, CEN, or HET domains. The nucleolus, seen as the large grey spherical bead in Fig. 6, strongly attracts the HET beads restraining their positioning to the “top” of the nucleus and clustering them, effectively polarizing the interphase chromatin.

The model employs four well-established major classes of TADs (Active, Null, PcG, and HP1) identified previously [59] based on their epigenetic signatures [107] and biological functions. Following bead–TAD equivalence in the model, it has four corresponding bead types. Each bead/TAD type is characterized by its own interaction well depth parameter $\epsilon _t$ for attractive interactions between beads of the same type. Interactions between beads of different types are not type-specific and are characterized by a single well-depth parameter $\epsilon _g$. The beads corresponding to TADs that contain lamina-associated domains (LADs) [41, 43]—L-TADs—can attractively interact with the NE. This L-TAD–NE affinity can temporally confine L-TADs at the NE. The interaction parameters of the model are tuned to reproduce: (i) the average experimental fraction of LADs confined to the NE, 25% [41] and (ii) the experimental TAD–TAD contact probability (Hi–C) map [59, 108] (Pearson’s correlation coefficient is 0.956). The model predicts highly dynamical distributions of the chromatin (both for control and lamins-depleted cells), which, after averaging, are in good agreement with the experimentally observed average density profiles of fruit fly chromatin [78]. As in the experiment, the chromatin density distribution in the model lamins-depleted nuclei shows a substantial shift of the chromatin away from the NE, compared to the distribution in control nuclei, accompanied by a large increase of the density in the central nucleus region, see Figure S2 in Additional file 1. This shift is also noticeable in Fig. 6, bottom row. The model predicts that both the shift of the chromatin from the NE and the increase of the chromatin density in the central region of lamins-depleted nuclei are sensitive to the strength of attractive TAD–TAD interactions. Eliminating the attraction between all the TADs in the model lamins-depleted nuclei, that is making it akin to a Gaussian chain, leads to about a factor of two increase of the chromatin density in the NE contact layer (within 0.2 $\mu$m of the NE, see below). The model also correctly reproduces the experimentally observed changes of average radial positioning of individual cytological regions (22A, 36C, and 60D) explored previously [46] in the lamins-depleted cells, Figure S1 in Additional file 1. The model is validated against multiple features of chromatin structure from several experiments not used in model development. Among model’s predictions is a correlation between the probability of a TAD to be near the NE (within 0.4 $\mu$m layer adjacent to the NE) and the local linear density of L-TADs along the chromatin chain [45] in the control nuclei.

The probability of a TAD being in contact with the NE is calculated as the probability of the center of the corresponding bead being within 0.2 $\mu$m spherical layer adjacent to the NE (grey “contact zone” in Fig. 6). The thickness of this layer is barely larger than the radius of the largest TAD (0.19 $\mu$m) and corresponds to the average TAD diameter in the nucleus model used. To calculate the probabilities, we analyzed 6 trajectories of model nuclei, covering all of the different nucleus chromatin topologies mentioned above. Each trajectory contains $400\times 10^3$ snapshots (chromatin configurations), and corresponds to approximately 11 h of nucleus time.

### Binning of TADs based on their probabilities to be in contact with the NE

The TADs are grouped into $K=6$ bins, according to the probability of each TAD in control cells being in contact with the NE. For 1169 TADs, Fig. 3 (A and D), bins #1–5 contain 195 TADs each, whereas bin #6 has 194 TADs. For 350 L-TADs, Fig. 3 (C and F), bins #1–5 contain 58 L-TADs each, whereas bin #6 has 60 L-TADs. For 819 NonL-TADs, Fig. 3 (B and E), bins #1–5 contain 137 NonL-TADs each, whereas bin #6 has 134 NonL-TADs.

The number of bins, *K*, is determined by a reasonable balance between two opposing requirements. On the one hand, enough bins are needed to identify a trend in transcription level changes as a function of stochastic proximity to the NE, but too many bins would result in fewer data points per bin, $\frac{1169}{K}$, leading to a higher standard error of the mean transcription level (s.e.m.) per bin. Requiring the error bar for each bin to be no greater than 20% of the bin height, that is s.e.m. $\le 0.2$ of the mean $\textrm{RPKMT}$ value per bin, we arrive at $K=6$ bins, which we argue is still large enough to discern meaningful trends of interest to us here.

### Supplementary information

**Additional file 1:** **Figure S1**. Probabilities of TADs (LAD containing TADs (L-TADs) and TADs not containing LADs (Non-L-TADs) in control nucleus model, and all TADs in lamins-depleted nucleus model) to be in contact with the NE (to be within 0.2 μm from the NE). Null L-TAD #15 (in control and lamins-depleted nuclei), analyzed in [1] as cytological region 22A, is marked by yellow circles. Null L-TAD #120 (in control and lamins-depleted nuclei), analyzed in [1] as cytological region 36C, is marked by red triangles. PcG L-TAD #435 (in control and lamins-depleted nuclei), analyzed in [1] as cytological region 60D, is marked by orange squares. **Figure S2**. **Left panel**: Computed chromatin density averaged over the spherical layers as a function of the radial distance from the nucleus center in control nuclei (top) and in lamins-depleted nuclei (bottom). The radius of the nucleus is 2 μm. **Right panel**: Experimental mean chromatin radial density in the equatorial plane of the nucleus of the proventriculus. For illustration only, the azimuthal dependence of the density is averaged out to produce a schematic that shows only the radial density profile. The density is inferred from relative fluorescence intensity, as detailed in Ref. [2]. Specifically, 21 equally spaced experimental data points are taken from **Fig. S3** (Group 1, bottom panel) of Ref. [2] and then interpolated using a linear interpolation process, yielding 201 equally spaced data points plotted in the figure. The radial position of the mean chromatin density is measured from the nuclear center to the periphery (0% - 100%). **Table S1**. A numerical simulation of gene activity with noise. Here, GC and GK are uniformly distributed random variables on the interval [0,1]. A total of 2N = 2000 random numbers were generated for each trial, and ratios of two sequential random numbers were computed and averaged over all N pairs. Each trial starts with an independent seed to initiate the random number generator Math.random() , as implemented in Java 1.16.4. **Figure S3**. (**A**) Scatter plot shows a weak negative correlation between the expression of genes in TADs (in RPKMTL) and the probability of TAD to be found in contact with the NE (i.e. to be found within 0.2 μm layer near the NE) in the control nuclei. (**B**) Scatter plot shows essentially no correlation between the TAD expression (in RPKMTL) and the probability of TAD being found in contact with the NE in the lamins-depleted nuclei. The Spearman, and Pearson correlation coefficients, their two-sided p-values (p), and linear regression lines (red) are shown. **Figure S4**. Dependencies of bin averaged TAD transcription levels (in RPKMTL) on the probability of TADs in the bin to be in contact with the NE. The binning of TADs is based on TAD-NE contact probabilities in control cells for each set (selection) of TADs. Solid bars: control cells. Empty bars: lamins-depleted cells. The same set of TADs per bin is used in the control and lamins-depleted cells. Error bars are s.e.m. (standard error of the mean). **Left panels**: (**A**) all TADs; (**B**) TADs not containing LADs (NonL-TADs); and (**C**) TADs containing LADs (L-TADs). In the left panels only, the positions of the empty bins (lamins-depleted cells) along the x-axis are deliberately kept unchanged to facilitate visual comparison with the heights of the corresponding bins for control cells. Right panels show only lamins-depleted cells: (**D**) all TADs; (**E**) TADs not containing LADs (NonL-TADs); and (**F**) TADs containing LADs (L-TADs). A clear shift of the average TAD positions away from the NE is evident. **Figure S5**. The metric of transcription activity in TADs, RPKMTL, is consistent with the epigenetic classes of TADs identified previously [3]. Median transcription level (in RPKMTL) in Active TADs (n=494) is at least 2 times greater than those of other epigenetic TAD classes, such as HP1/centromeric (n=52), Null (n=492), and PcG (n=131) (panel A, dashed lines). The medians (dashed lines) along with the means (solid boxes) demonstrate consistency with the data in Figure 3C of Ref. [3], reproduced in the panel B, which show the median gene transcription levels within each epigenetic class of TADs. The dynamic model of fruit fly nucleus employs the partitioning of the genome into TADs and their epigenetic classes, introduced in Ref. [3]. Error bars are s.e.m. (standard error of the mean). **Figure S6**. Distribution of TADs by types such as Active L-TADs (n=54), Active NonL-TADs (n=440), HP1/centromeric L-TADs (n=34), Null L-TADs (n=228), Null NonL-TADs (n=264), PcG L-TADs (n=50), and PcG NonL-TADs (n=81) in control (**A**) and lamins-depleted (**B**) cells. The average gene expression (black horizontal lines) in L-TADs for each type is lower than those of NonL-TADs. The red horizontal lines are the median gene expression values in TADs for each type. **Figure S7**. Distribution of TADs in bins by epigenetic classes (Null, Active, PcG, and HP1/centromeric). **A**, **B**, and **C** The red and black horizontal lines are the median and mean gene expression values (in RPKMT) in each bin, respectively. **D**, **E**, and **F** Comparison of the number of TADs of each epigenetic class in each bin. For NonL-TADs (control cells), the number of Active TADs is greater than those of other epigenetic classes in each bin. In contrast, for L-TADs (control cells), the number of Null TADs is greater than those of other epigenetic class in each bin. Positions of TADs along the horizontal axis in bins are not to scale.

**Additional file 2: Movie.** Time evolution of the model interphase chromosomes of fruit fly at TAD resolution. The corresponding biological time interval is approximately one minute, from t = 0. Chromosome arms: 2L (purple), 2R (orange), 3L (yellow), 3R (green), X (blue) and 4(cyan). The centromeres and telomeres are shown by black and red spheres, respectively.

**Additional file 3: Movie.** Time evolution of the model interphase chromosomes of fruit fly at TAD resolution. The corresponding biological time interval is approximately one hour, from t = 0. Chromosome arms: 2L (purple), 2R (orange), 3L (yellow), 3R (green), X (blue) and 4(cyan). The centromeres and telomeres are shown by black and red spheres, respectively.

**Additional file 4: Movie.** Time evolution of the model interphase chromosomes of fruit fly at TAD resolution. The corresponding biological time interval is approximately eleven hours, from t = 0. Chromosome arms: 2L (purple), 2R (orange), 3L (yellow), 3R (green), X (blue) and 4(cyan). The centromeres and telomeres are shown by black and red spheres, respectively.
