# Supplementary material for: The probability of chromatin to be at the nuclear lamina has no systematic effect on its transcription level in fruit flies
Source: Epigenetics Chromatin. 2024 May 6;17:13. doi: 10.1186/s13072-024-00528-8 (PMC11071202; doi:10.1186/s13072-024-00528-8)
Supplement: Supplementary file 1 — Additional file 1: Figure S1. Probabilities of TADs (LAD containing TADs (L-TADs) and TADs not containing LADs (Non-L-TADs) in control nucleus model, and all TADs in lamins-depleted nucleus model) to be in contact with the NE (to be within 0.2 μm from the NE). Null L-TAD #15 (in control and lamins-depleted nuclei), analyzed in [1] as cytological region 22A, is marked by yellow circles. Null L-TAD #120 (in control and lamins-depleted nuclei), analyzed in [1] as cytological region 36C, is marked by red triangles. PcG L-TAD #435 (in control and lamins-depleted nuclei), analyzed in [1] as cytological region 60D, is marked by orange squares. Figure S2. Left panel: Computed chromatin density averaged over the spherical layers as a function of the radial distance from the nucleus center in control nuclei (top) and in lamins-depleted nuclei (bottom). The radius of the nucleus is 2 μm. Right panel: Experimental mean chromatin radial density in the equatorial plane of the nucleus of the proventriculus. For illustration only, the azimuthal dependence of the density is averaged out to produce a schematic that shows only the radial density profile. The density is inferred from relative fluorescence intensity, as detailed in Ref. [2]. Specifically, 21 equally spaced experimental data points are taken from Fig. S3 (Group 1, bottom panel) of Ref. [2] and then interpolated using a linear interpolation process, yielding 201 equally spaced data points plotted in the figure. The radial position of the mean chromatin density is measured from the nuclear center to the periphery (0% - 100%). Table S1. A numerical simulation of gene activity with noise. Here, GC and GK are uniformly distributed random variables on the interval [0,1]. A total of 2N = 2000 random numbers were generated for each trial, and ratios of two sequential random numbers were computed and averaged over all N pairs. Each trial starts with an independent seed to initiate the random number generator Math.random() , as im [file 13072_2024_528_MOESM1_ESM.pdf]

RESEARCH

Supplementary Information: The probability of chromatin to be at the nuclear lamina has no systematic effect on its transcription level in fruit flies

Alexander Y. Afanasyev[1†], Yoonjin Kim[2†], Igor S. Tolokh[2†], Igor V. Sharakhov[3] and Alexey V. Onufriev[2,4,5*]

*Correspondence: alexey@cs.vt.edu
[2]Department of Computer Science, Virginia Polytechnic Institute and State University, Blacksburg, VA 24061, USA
[4]Department of Physics, Virginia Polytechnic Institute and State University, Blacksburg, VA 24061, USA
[5]Center for Soft Matter and Biological Physics, Virginia Polytechnic Institute and State University, Blacksburg, VA 24061, USA
Full list of author information is available at the end of the article
†These authors contributed equally to this work

Distributions of TADs in the Drosophila nucleus

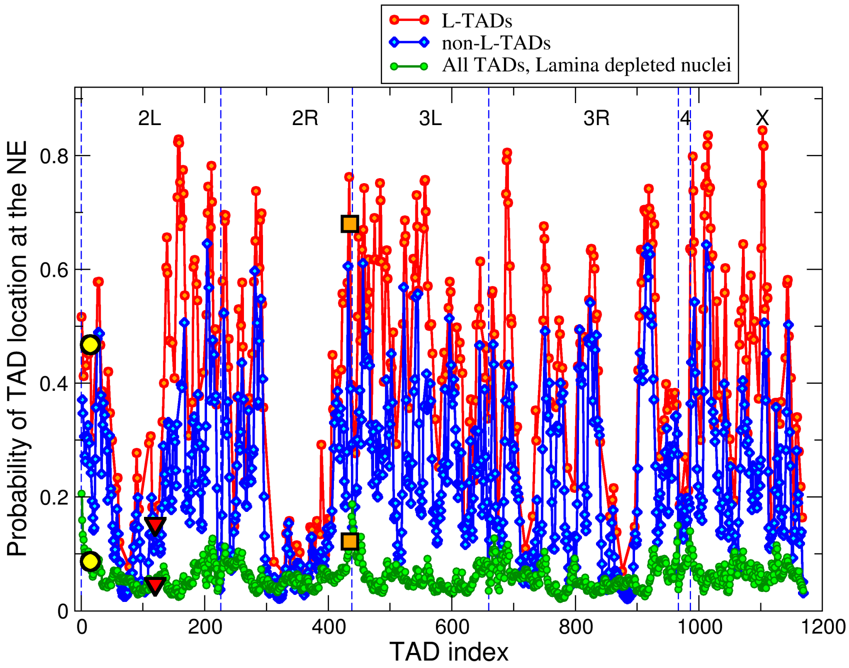

Figure S1 Probabilities of TADs (LAD containing TADs (L-TADs) and TADs not containing LADs (Non-L-TADs) in control nucleus model, and all TADs in lamins-depleted nucleus model) to be in contact with the NE (to be within 0.2 μm from the NE). Null L-TAD #15 (in control and lamins-depleted nuclei), analyzed in [1] as cytological region $22A$, is marked by yellow circles. Null L-TAD #120 (in control and lamins-depleted nuclei), analyzed in [1] as cytological region $36C$, is marked by red triangles. PcG L-TAD #435 (in control and lamins-depleted nuclei), analyzed in [1] as cytological region $60D$, is marked by orange squares.

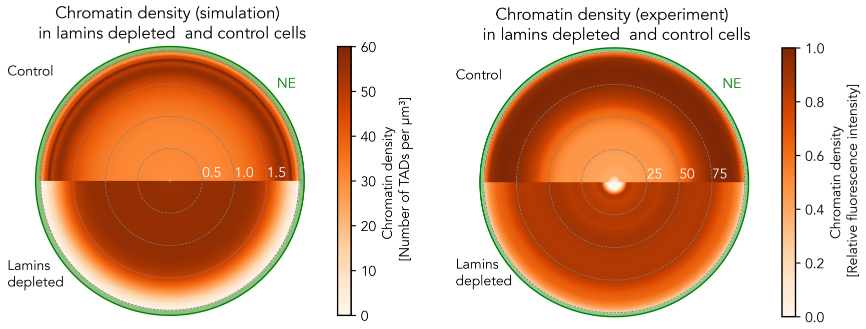

Figure S2 Left panel: Computed chromatin density averaged over the spherical layers as a function of the radial distance from the nucleus center in control nuclei (top) and in lamins-depleted nuclei (bottom). The radius of the nucleus is 2 μm. **Right panel**: Experimental mean chromatin radial density in the equatorial plane of the nucleus of the proventriculus. For illustration only, the azimuthal dependence of the density is averaged out to produce a schematic that shows only the radial density profile. The density is inferred from relative fluorescence intensity, as detailed in Ref. [2]. Specifically, 21 equally spaced experimental data points are taken from Fig. S3 (Group 1, bottom panel) of Ref. [2] and then interpolated using a linear interpolation process, yielding 201 equally spaced data points plotted in the figure. The radial position of the mean chromatin density is measured from the nuclear center to the periphery (0% - 100%).

Table S1 A numerical simulation of gene activity with noise. Here, G^C and G^K are uniformly distributed random variables on the interval [0,1]. A total of $2N = 2000$ random numbers were generated for each trial, and ratios of two sequential random numbers were computed and averaged over all N pairs. Each trial starts with an independent seed to initiate the random number generator `Math.random()` , as implemented in Java 1.16.4.

trial #	1	2	3	4	5
$\langle G^C/G^K \rangle$	4.45	3.64	6.40	6.68	2.82

Comparing averages and ratios of gene expression levels

We argue here that, counter-intuitively, the use of ratios of gene expression levels to characterize possible differences in transcription activities between two sets of genes (e.g., knockdown vs. control) can lead to unintended biases due to inherent noise in the data. For the sake of argument, consider a simplified case of two sets C and K, of N genes in each set, each gene having the same inherent transcription level in both sets. Due to the inevitable stochasticity of gene expression, especially relevant at low levels, and because of experimental uncertainty, the actual measurement of each gene activity will be a random variable G_i^C (and G_i^K) with some distribution, here assumed identical for all genes. For the sake of argument, assume this distribution to be uniform on the gene activity interval from 0 to 1. Obviously, in this case the mean expression level $\langle G_i \rangle$ of each gene is exactly 1/2, the activity averaged over each gene set $\langle G^C \rangle = \langle G^K \rangle = \frac{1}{N} \sum_i G_i = 1/2$. That is if one uses transcription activity averages to compare two sets of genes, their activities are the same, as expected. The situation is different if one attempts to use $\frac{G_i^C}{G_i^K}$ to make the comparison, *e.g.* to evaluate the effect of a knockdown. Note that, in general, the average of a ratio does not equal the ratio of the averages; a numerical example is shown in Table S1. The intuitive rationale for the effect is as follows: a deviation of the denominator down from its mean value causes a larger increase of the fraction than does the decrease of the fraction caused by the same size deviation of the denominator up from its mean. Rigorous analysis shows that in the case of the uniform distribution on [0 1] interval, the mean of the ratio diverges (logarithmically), which explains the large variation of the mean ratio from one trial (”experiment”) to another. Thus, each independent set of measurements can bring about a different outcome in terms of the ratio of the gene activities, Table S1.

The key conclusions remain valid to another metric of transcription activity in TADs

We propose another metric of transcription activity in TADs – RPKMTL (number of Reads mapped to all genes in a TAD per kilobase of TAD length per Million reads mapped to all TADs). Unlike RPKMT, RPKMTL uses the length of a TAD to obtain the average transcription activity at TAD resolution. RPKMTL characterizes an average expression of all genes in a TAD and is defined as:

$$\text{RPKMTL} = \frac{10^6 \times \text{Reads mapped to genes in a TAD}}{\text{Total mapped reads} \times \text{TAD length in kb}} \tag{1}$$

For two replicates (rep1 and rep2) from published RNA-seq data [1], the transcription activity metric, defined in Eq. 1, is calculated as:

$$\text{RPKMTL} = \frac{10^6 \times (\text{Sum of reads of rep1 and rep2 mapped to genes in a TAD})}{(\text{Total mapped reads of rep1 and rep2}) \times \text{TAD length in kb}} \tag{2}$$

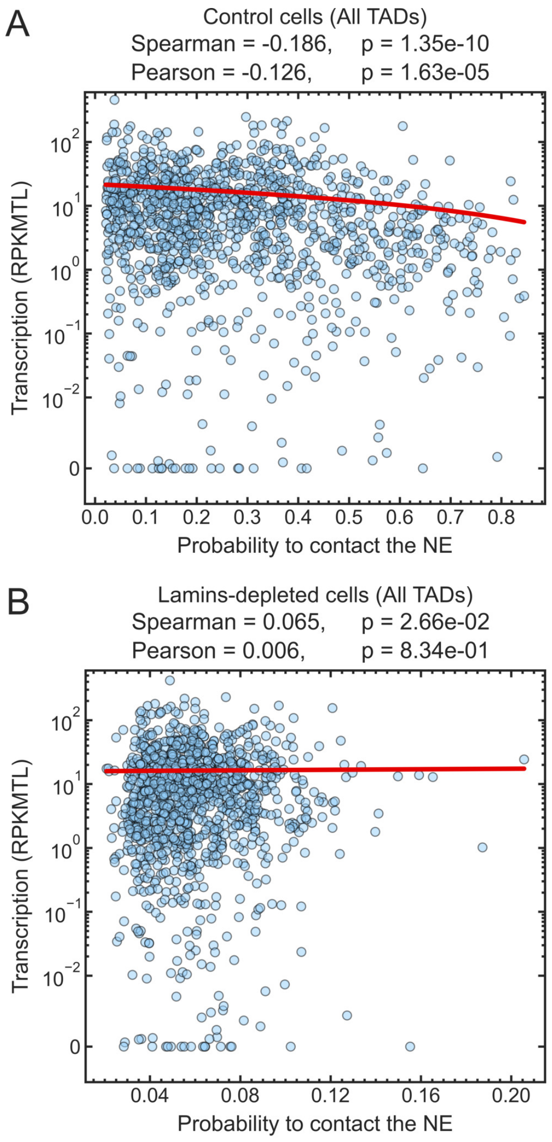

Figure S3 **(A)** Scatter plot shows a weak negative correlation between the expression of genes in TADs (in RPKMTL) and the probability of TAD to be found in contact with the NE (i.e. to be found within 0.2 μm layer near the NE) in the control nuclei. **(B)** Scatter plot shows essentially no correlation between the TAD expression (in RPKMTL) and the probability of TAD being found in contact with the NE in the lamins-depleted nuclei. The Spearman, and Pearson correlation coefficients, their two-sided p-values (p), and linear regression lines (red) are shown.

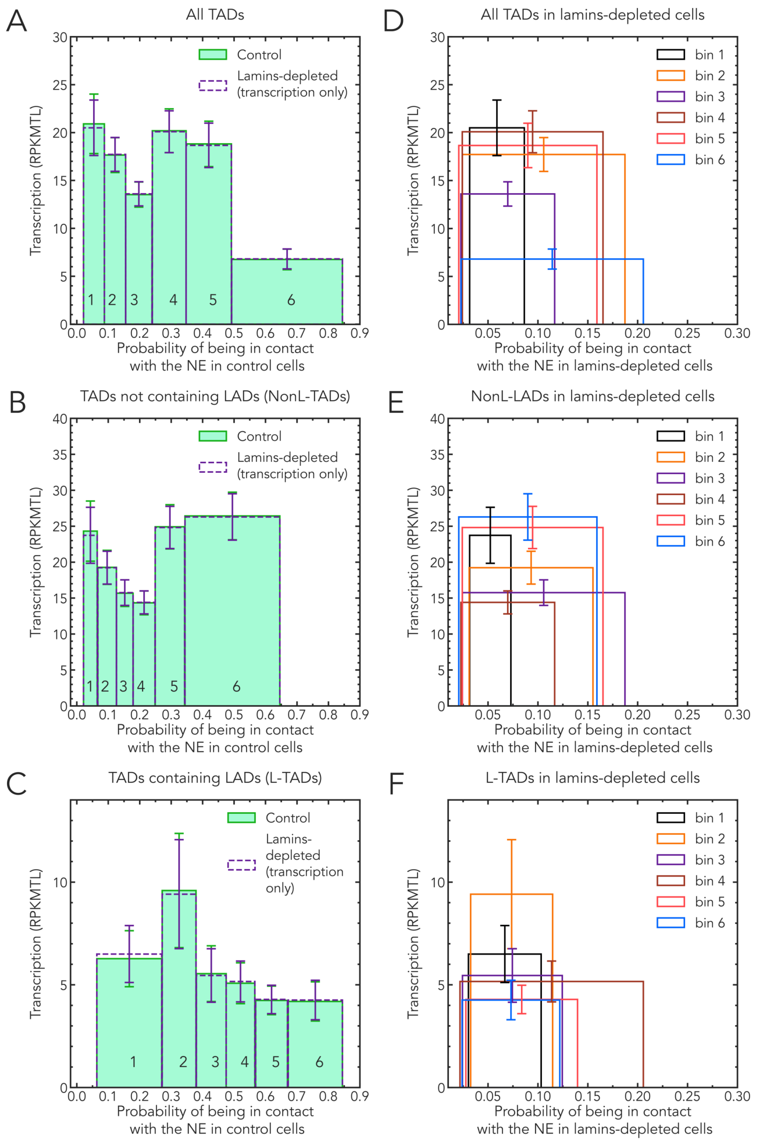

Figure S4 Dependencies of bin averaged TAD transcription levels (in RPKMTL) on the probability of TADs in the bin to be in contact with the NE. The binning of TADs is based on TAD-NE contact probabilities in control cells for each set (selection) of TADs. **Solid bars: control cells. Empty bars: lamins-depleted cells.** The same set of TADs per bin is used in the control and lamins-depleted cells. Error bars are s.e.m. (standard error of the mean). **Left panels: (A)** all TADs; **(B)** TADs not containing LADs (NonL-TADs); and **(C)** TADs containing LADs (L-TADs). In the left panels only, the positions of the empty bins (lamins-depleted cells) along the x-axis are deliberately kept unchanged to facilitate visual comparison with the heights of the corresponding bins for control cells. **Right panels show only lamins-depleted cells: (D)** all TADs; **(E)** TADs not containing LADs (NonL-TADs); and **(F)** TADs containing LADs (L-TADs). A clear shift of the average TAD positions away from the NE is evident.

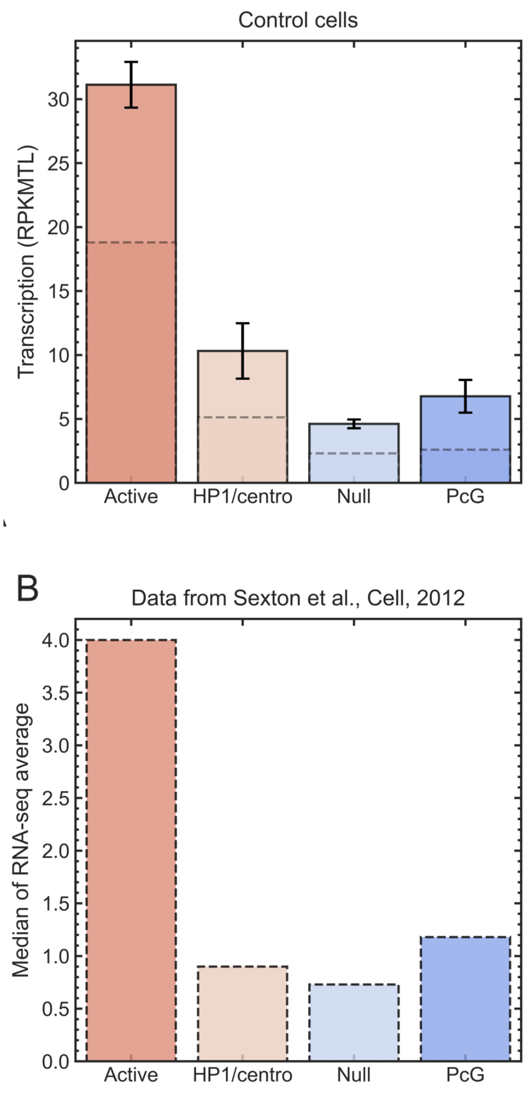

Figure S5 The metric of transcription activity in TADs, RPKMTL, is consistent with the epigenetic classes of TADs identified previously [3]. Median transcription level (in RPKMTL) in Active TADs (n=494) is at least 2 times greater than those of other epigenetic TAD classes, such as HP1/centromeric (n=52), Null (n=492), and PcG (n=131) (**panel A, dashed lines**). The medians (dashed lines) along with the means (solid boxes) demonstrate consistency with the data in Figure 3C of Ref. [3], reproduced in the **panel B**, which show the median gene transcription levels within each epigenetic class of TADs. The dynamic model of fruit fly nucleus employs the partitioning of the genome into TADs and their epigenetic classes, introduced in Ref. [3]. Error bars are s.e.m. (standard error of the mean).

Distribution of TADs by types and epigenetic classes

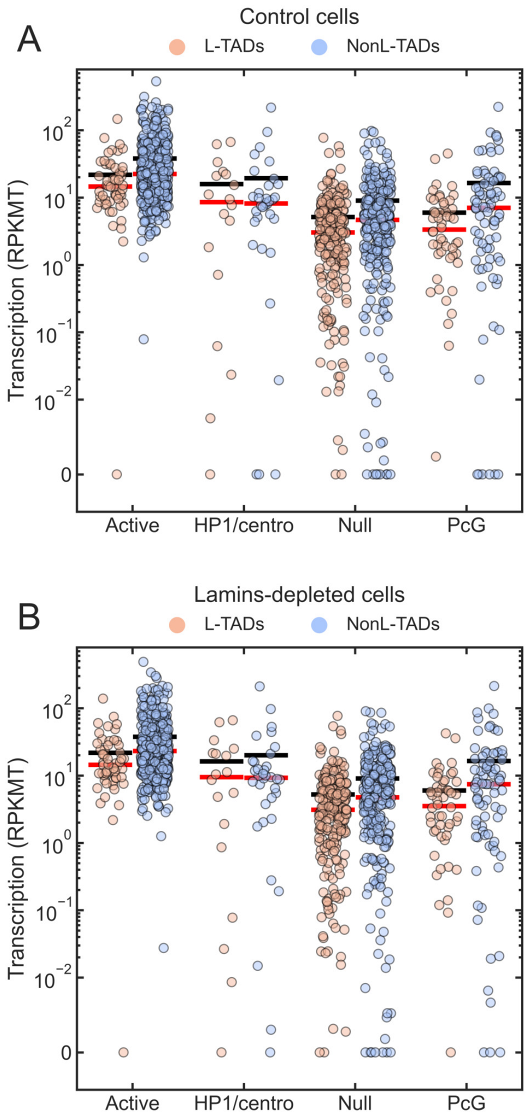

Figure S6 Distribution of TADs by types such as Active L-TADs (n=54), Active NonL-TADs (n=440), HP1/centromeric L-TADs (n=34), Null L-TADs (n=228), Null NonL-TADs (n=264), PcG L-TADs (n=50), and PcG NonL-TADs (n=81) in control (**A**) and lamins-depleted (**B**) cells. The average gene expression (black horizontal lines) in L-TADs for each type is lower than those of NonL-TADs. The red horizontal lines are the median gene expression values in TADs for each type.

A note on bins #3 and #4 in Figure 3B of the main text.

Below is a possible explanation for why the average transcription levels (in RPKMT) of NonL-TADs in bins #3 and #4 in Figure 3B, corresponding to the 0.15–0.28 probabilities of NonL-TADs to be in contact with the NE, are relatively low. Here, we compared the fractions of different epigenetic classes of TADs in each of the six bins in Figure 3B. Bins #3 and #4 demonstrate a reduced number of Active TADs, which have a much higher average transcription level compared to the other three (non-Active) epigenetic classes of TADs (see Figure 5), and increased fractions of non-Active TADs, see Figure S7E below. In contrast, bin #5 and bin #6 (relatively high average RPKMT levels in Figure 3B) have higher fractions of Active TADs relative to other bins (see Figure S7E).

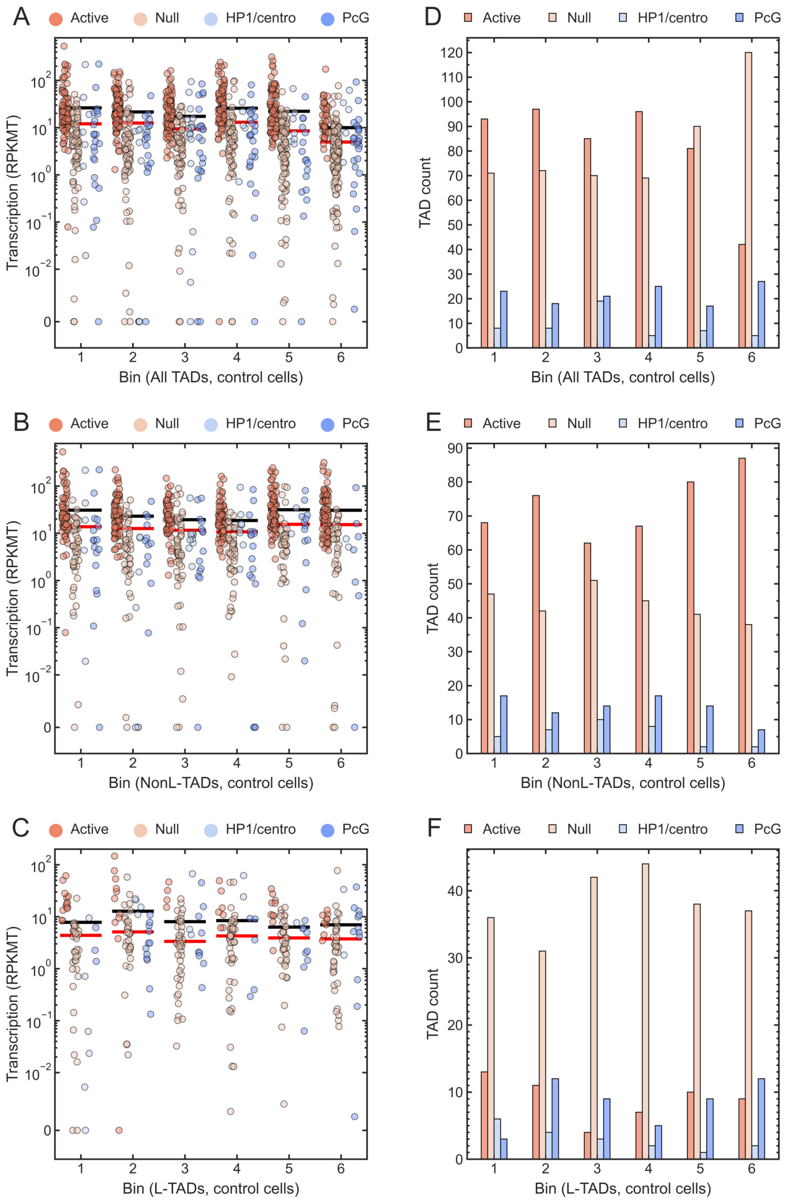

Figure S7 Distribution of TADs in bins by epigenetic classes (Null, Active, PcG, and HP1/centromeric). **(A, B, and C)** The red and black horizontal lines are the median and mean gene expression values (in RPKMT) in each bin, respectively. **(D, E, and F)** Comparison of the number of TADs of each epigenetic class in each bin. For NonL-TADs (control cells), the number of Active TADs is greater than those of other epigenetic classes in each bin. In contrast, for L-TADs (control cells), the number of Null TADs is greater than those of other epigenetic class in each bin. Positions of TADs along the horizontal axis in bins are not to scale.

Acknowledgements
A.V.O. thanks Andrei Onufriev for implementing and running the numerical example supporting the gene activity ratio analysis.

Author details
[1]Department of Biomedical Engineering and Mechanics, Virginia Polytechnic Institute and State University, Blacksburg, VA 24061, USA. [2]Department of Computer Science, Virginia Polytechnic Institute and State University, Blacksburg, VA 24061, USA. [3]Department of Entomology, Virginia Polytechnic Institute and State University, Blacksburg, VA 24061, USA. [4]Department of Physics, Virginia Polytechnic Institute and State University, Blacksburg, VA 24061, USA. [5]Center for Soft Matter and Biological Physics, Virginia Polytechnic Institute and State University, Blacksburg, VA 24061, USA.

References

1. Ulianov, S.V., Doronin, S.A., Khrameeva, E.E., Kos, P.I., Luzhin, A.V., Starikov, S.S., Galitsyna, A.A., Nevasheva, V.V., Ilyin, A.A., Flayamer, I.M., Mikhaleva, E.A., Logacheva, M.D., Gelfand, M.S., Chertovich, A.V., Gavrilov, A.A., Razin, S.V., Sheveloyov, Y.Y.: Nuclear lamina integrity is required for proper spatial organization of chromatin in drosophila. Nat Commun **10** (2019). doi:10.1038/s41467-019-09185-y
2. Bondarenko, S.M., Sharakhov, I.V.: Reorganization of the nuclear architecture in the drosophila melanogaster lamin b mutant lacking the CaaX box. Nucleus **11**(1), 283–298 (2020). doi:10.1080/19491034.2020.1819704
3. Sexton, T., Yaffe, E., Kenigsberg, E., Bantignies, F., Leblanc, B., Hoichman, M., Parrinello, H., Tanay, A., Cavalli, G.: Three-dimensional folding and functional organization principles of the drosophila genome. Cell **148**(3), 458–472 (2012). doi:10.1016/j.cell.2012.01.010
